# Effect of early “quilting” sutures on morbidity in postpartum hemorrhage

**DOI:** 10.1002/ijgo.12700

**Published:** 2018-11-14

**Authors:** Werner Stein, Ludwig Spätling

**Affiliations:** ^1^ Department of Obstetrics and Gynecology Klinikum Fulda Academic Teaching Hospital of the University of Marburg Fulda Germany

**Keywords:** Cesarean, Compression sutures, Hysterectomy, Postpartum hemorrhage, Quilting sutures

## Abstract

**Objective:**

To examine whether “quilting” sutures are safe and effective in preventing hysterectomy among women with postpartum hemorrhage (PPH) and whether early application might reduce the incidence of transfusion.

**Methods:**

Data were reviewed from women treated via quilting sutures after cesarean delivery at a university hospital between 2007 and 2016. Objective and subjective data were collected by analyzing medical records and performing telephone interviews. To observe trends during the study period, data from the first 50% of women treated were compared with those from the second 50%.

**Results:**

Overall, 26 cesareans with quilting sutures were performed. Two hysterectomies could not be avoided. During 2012–2106, 18 quilting sutures were performed as compared with 8 in 2007–2011, pointing to a more liberal indication. Intensive care was required twice as frequently among the first 13 procedures than among the second 13 procedures (10 vs 5, respectively). A similar observation was made for the use of blood transfusions or clotting activation (9 vs 4, respectively). Three women who desired to have a child subsequently delivered a newborn.

**Conclusion:**

Quilting sutures were found to be a safe and simple technique to prevent hysterectomies in PPH. Morbidity was reduced when the decision to perform sutures was taken early.

## Abstract 2

Performing quilting sutures early in postpartum hemorrhage was found to prevent hysterectomy and reduce morbidity.

## 1 INTRODUCTION

Postpartum hemorrhage (PPH) remains a central cause of maternal mortality.^1^ The treatment methods of B‐Lynch et al.^2^ and Pereira et al.^3^ are relatively complicated. In addition, compression techniques such as the Bakri balloon^4^ are not available everywhere, are expensive, and hinder the normal contraction of the recovering myometrium.

At the Academic Teaching Hospital of the University of Marburg, Fulda, Germany, a backstitch quilting technique (hereafter “quilting” sutures) for treatment of PPH was developed in 2007 and the first results of the approached were published in 2012.^5^, ^6^ The aim of the present study was to evaluate the safety and tolerability of the procedure by review and follow‐up interview of women treated by quilting sutures over a 10‐year period, and to determine whether the approach warrants general recommendations.

## 2 MATERIALS AND METHODS

The present descriptive retrospective study evaluated the medical histories and clinical records of all women treated by using quilting sutures between January 1, 2007, and December 31, 2016, at the Department of Obstetrics and Gynecology, Klinikum Fulda, Fulda, Germany. Ethics Committee approval was not needed because the retrospective study design relied on patient records. Because PPH is unpredictable, the women were not asked to give consent prior to surgery. All women who were interviewed by telephone consented to take part in the study.

Quilting sutures were used on all patients with PPH after cesarean when application of the myometrium‐contracting substances oxytocin (Hexal AG, Holzkirchen, Germany) and Sulprostone (Nalador; Jenapharm, Jena, Germany). Starting at the top of the uterus continuing down to the cervical segment, horizontally placed U‐sutures were used to fix the anterior to the posterior wall. Up to 12 size‐1 absorbable sutures (Vicryl; Ethicon, Bridgewater, NJ, USA) were applied to compress the uterus completely. Rapidly absorbable suture material was avoided because it has a lower tensile strength. In the absence of straight needles, the technique employed curved needles, which curved from 7.5 cm to 9 cm (DS 95; Serag Wiessner, Naila, Germany). The procedure has been demonstrated in a short film.^6^

The quilting sutures procedure was initially indicated and performed by the director of the clinic. As they gained in experience, senior physicians were instructed in the technique and then also performed the procedure. As a result, the change in management policy toward a more rapid decision to use quilting sutures was a continuous process.

To distinguish trends, data were compared between the first 50% (first group) and the second 50% (second group) of women treated by quilting sutures. In the second group, quilting sutures were used more liberally, that is, immediately after failure of medical treatment and before the onset of massive loss of blood and clotting factors.

To obtain follow‐up data, women were contacted by telephone and asked to participate in a short interview, including complications since hospital discharge, state of health, and desire for children. For women treated with quilting sutures within 6 months of the start of evaluation, the interviews regarding personal condition and state of health were conducted by the same obstetrician.

Data were compiled using Microsotft Excel (Microsoft, Redmond, WA, USA). The data were summarized by using simple statistics. Because they were not normally distributed, they were presented as number, mean, and median (range).

## 3 RESULTS

During the 10‐year study period, quilting sutures were used to treat PPH among 26 women. Eight procedures were carried out in the first 5‐year period (2007–2011), as compared with 18 in the second 5‐year period (2012–2016).

The biometric data, gravidity, and parity of the study women were similar between the first 13 women and the second 13 women treated (Table 1). Eleven of the 26 study women had additional obstetric and clinical characteristics. Among the first 13 women, these included previous cesarean (n=2), complete occlusion of cervical os plus cerclage (n=1), diabetes mellitus (n=1,) history of pulmonary edema (n=1), history of spontaneous late abortion (n=1), history of induced abortion (n=1). Among the second 13 women, characteristics included previous cesarean (n=2), history of transfusion because of atony (n=1), pulmonary embolism (n=1), HELLP (hemolysis, elevated liver enzyme levels, and low platelet levels) syndrome (n=1), factor V Leiden mutation (n=1), anemia because severe iron deficiency (n=1), pulmonary embolism (n=1), Couvelaire uterus (n=1), and placenta increta (n=1).

**Table 1:** Characteristics of the study patients.

Characteristic	All women (n=26)		1st group (n=13)		2nd group (n=13)	
	Mean	Median (range)	Mean	Median (range)	Mean	Median (range)
Age, y	30.5	29 (20–40)	30.8	31 (20–40)	30.2	29 (21–37)
Length, cm	166	165 (158–175)	167	166 (162–175)	166	166 (158–170)
Weight before, kg	69	65 (47–112)	70.3	62.5 (47–112)	67.5	65 (51–100)
Weight at delivery, kg	81.5	77 (56–128)	84	77 (57–128)	79.1	75 (56–118)
Gravidity	2.1	1 (1–5)	2.2	2 (1–4)	2	1 (1–5)
Parity	1.4	0 (0–4)	0.6	1 (0–1)	0	0 (0–4)

The indications for cesarean were fetal distress (n=3), twin gestation (n=3), preterm rupture of membranes (n=3), dystocia (n=2), placental abruption (n=2), uterine rupture (n=1), severe pre‐eclampsia (n=1), amnion infection (n=1), placenta previa (n=1), complete occlusion of cervical os plus cerclage (n=1), placenta accreta (n=2) in the first group; and twin gestation (n=4), placental abruption (n=4), preterm rupture of membranes (n=3), threatening uterine rupture (n=2), dystocia (n=2), HELLP syndrome (n=1), insertion velamentosa (n=1), anhydramnion (n=1), intrauterine death (multiple trauma) (n=1), intrauterine growth retardation (n=1) in the second group. Note that some women had more than one indication.

In the second group, two cases were complicated by further problems not related to PPH. One case involved polytrauma, including placental abruption owing to a car accident that resulted in intrauterine fetal death; the patient received a massive blood transfusion. In the second case, pulmonary embolism occurred during the cesarean and was diagnosed 1 day later.

Regarding the characteristics of the cesarean deliveries, nine women had induction of labor, mostly by misoprostol (Table 2). For 13 women, either transfusion or clotting activation was necessary (Table 3). Most of these women were in the first group (Table 3). The greater number of manual compressions performed before applying quilting sutures in the first group (n=7) as compared with the second group (n=1) demonstrated the move toward more frequent application of the method.

**Table 2:** Characteristics of delivery among the study patients.

Characteristic	All women (n=26)		1st group (n=13)		2nd group (n=13)	
	Value^a^	Median (range)	Value^a^	Median (range)	Value	Median (range)
Induction of labor	9		7		2	
Misoprostol	8		6		2	
Prostaglandin E_2_ gel	1		1			
Oxytocin during delivery	4		2		2	
Gestational age, wk^+d^	35^+1^	36^+2^ (24^+0^–42^+0^)	37^+4^	37^+6^ (28^+5^–41^+4^)	32^+6^	32^+4^ (24^+0^–42^+0^)
Birthweight, g	2390	2630 (484–4200)	2824	2840 (1380–3650)	1981	2147 (484–4200)
Estimated blood loss, mL	1737	1500 (500–4500)	2140	2000 (1000–4500)	150	1000 (500–2000)
Transfusion/clotting factors administered	13		9		4	
Admitted to ICU	15		10		5	
Stay in ICU, h	29	20 (12–72)	24	20 (12–59)	39	48 (12–72)
Hysterectomy performed	2		0		2	
Manual uterus compression performed	8		7		1	
Stay at hospital, d	9.1	7 (4–26)	8.5	7 (4–14)	9.1	6 (5–26)
Hb before delivery, g/dL	11.6	11.7 (8.7–14.1)	11.9	11.6 (10.5–14.1)	11.2	12.0 (8.7–12.9)
Lowest Hb, g/dL	6.4	6.4 (3.0–9.4)	5.9	6.1 (3.0–9.0)	7.1	6.8 (5.4–9.4)
Hb at discharge, g/dL	8.9	8.9 (6.5–11.5)	8.7	8.1 (6.5–11.0)	9.3	9.1 (7.2–11.5)

Abbreviation: ICU, intensive care unit.

a Values are given as mean or absolute number.

**Table 3:** Details of coagulation factors and/or transfusion (n=13).

Factor	No. of women overall (n=13)	1st group (n=9)		2nd group (n=3)	
		Values^a^	Median (range)	Values^a^	Median (range)
Packed erythrocytes, units	10	18;2;4;10;6;8;2	6 (2–18)	2;16;2	2 (2–18)
Tramexanic acid, g	7	2;1;1;1;2	1 (1–2)	2;2	2 (2–2)
Fresh frozen plasma, units	5	14;4;10;4	7 (4–14)	12	12
Fibrinogen, g	4	4;2;4;4	4 (2–4)		
PCC (1500 IE)	2	1;1	1 (1–1)		
Packed thrombocytes, units	4	1;2;1	1 (1–2)	4	4
NovoSeven 5 mg^b^	1	1	1		

Abbreviations: IE, international unit; PCC, prothrombin complex concentrate.

a Values are given as absolute number.

b Novo Nordisk, Bagsvaerd, Denmark.

Overall, the number of women needing transfusion or intensive care in the second group was half that in the first group (3 vs 7 and 5 vs 10, respectively). The long mean stay in both hospital and the intensive care unit in the second group (9.1 days and 39 hours, respectively) was due the inclusion of a women with the lung embolism (n=1) and a car accident with multiple trauma (n=1) (Table 2). A hysteroscopy was performed for two women and showed no significant adhesions. Two hysterectomies were carried out. In the pulmonary embolism case, therapeutic anticoagulation was initiated and resulted in persistent bleeding; thus, the interdisciplinary team decided to perform a supracervical hysterectomy. For the other patient, it was not possible to stop PPH via the quilting sutures; thus, strictly speaking, the technique failed once (1/26; 4%).

In total, 14 women completed the follow‐up telephone interviews (Table 4). The responses demonstrated good recovery of the women. For women not on hormonal contraceptives, menstruation had restarted by 7 weeks postpartum. Amenorrhea and menstrual discomfort after quilting sutures was uncommon, and only one women reported a lack of vaginal discharge (Table 4). All women reported that they were pleased to have kept their uterus even if they did not desire a child. Three (21%) of the 14 women reported having had a child since the procedure. Where their personal situation permitted, all women desiring to have a child were able to do so.

**Table 4:** Data from follow‐up interviews among 14 study patients.^a^

Question	No. of women	Responses
Complications after hospital discharge	3	Fever, antibiotics (n=1); fever, curettage, antibiotics (n=1); spotting for 2 wk, hematoma (n=1)
Duration to feeling fit	14	Mean, 3.8 wk; median, 3 wk (range, 1–10 wk)
Vaginal discharge	14	None (n=1); minor (n=5); normal (n=7); intense (n=1)
Menstruation		
Duration until restarted	14	Mean, 7.3 wk; median, 8 wk (range, 3–12 wk)
No menstruation	4	Breastfeeding (n=2); polycystic ovaries (n=1); hormonal contraceptive (n=1)
Intensity	10	Less than before (n=6); same as before (n=1); more intense than before (n=3)
Discomfort	10	Less than before (n=6); same as before (n=1); more intense than before (n=3)
Children since cesarean		
Born	3	Born 3, 4, and 7 y after the procedure (3/14; 21%)
Desired	7	Desired children, but too close to last delivery (n=3); desired children, but undecided because of past delivery (n=2); desired children, but no partner (n=2)
Other comments	14	All women felt lucky to have kept their uterus even if they did not desire children

a Five women were not interviewed because the index cesarean was in the past 6 months; 15 women were contacted, but one (who had lung embolism and hysterectomy) declined to take part.

## 4 DISCUSSION

The present study indicates that the horizontal backstitch quilting sutures method is a safe, easy‐to‐perform, and easy‐to‐remember means of avoiding hysterectomy in cases of PPH. This is reflected by the increasing number and the earlier indication of quilting sutures before the women were affected by anemia and clotting problems. The decrease in morbidity and ease of handling have contributed to a reduction of fear of PPH on the delivery ward. Quilting sutures failed only once because of ongoing bleeding due to therapeutic anticoagulation.

Early application of quilting sutures before the onset of massive blood loss reduced the need for blood transfusion, especially the need for mass transfusion. Furthermore, the incidence of admission to the intensive care unit and prolonged hospitalization were considerably reduced.

In other techniques for treating PPH using compression sutures, infection (pyometria),^7^ ischemia (necrosis),^8^ and synechiae^9^ have been described. The lack of such complications in the present study could be due to the horizontal application of U‐stiches, which facilitates optimal oxygenation from both arteriae uterinae. Compression of a bigger volume of myometrium (e.g., square stiches, B‐Lynch, and Cho^10^) might hinder oxygenation. By using horizontal stiches, it may be possible to achieve earlier physiologic contraction and recovery of the myometrium. Note that in cases of PPH after vaginal delivery with extensive trauma of vaginal tissue at the study hospital, the “sumo‐compression” technique^11^, ^12^ is used when suturing is not effective.

The study has some limitations. First, it was not a prospective randomized clinical trial and does not provide level A evidence. Second, the number of cases was small, although the study period covered 10 years; however, designing and conducting studies of emergency cases is very difficult.

Although the study is retrospective, it is hoped that the results will motivate other surgeons to use quilting sutures in cases of PPH with the aim of reducing maternal morbidity and mortality. We recommend that, if oxytocin followed by prostaglandin administration fails to achieve appropriate myometrial contractility, quilting sutures should be promptly applied. Doctors should not wait for clinically relevant anemia or clotting anomalies to occur. Postoperative antibiotics are also recommended.

## AUTHOR CONTRIBUTIONS

WS contributed to performing the surgical procedures described, data collection, analyzing the data, and writing the manuscript. LS contributed to performing the surgical procedures described, analyzing the data, and writing the manuscript.

## CONFLICTS OF INTEREST

The authors have no conflicts of interest.
